# Predictive value of haematological indices on mortality in patients with empyema

**DOI:** 10.5937/jomb0-57112

**Published:** 2026-02-27

**Authors:** Ali Kutta Çelik, Abdikani Ali Salad, Okke§ Zortuk

**Affiliations:** 1 Department of Infectious Diseases and Clinical Microbiology Mogadishu-Somalia-Turkey Recep Tayyip Erdo an Training and Research Hospital, Mogadishu, Somalia; 2 Department of Thoracic Surgery Mogadishu-Somalia-Turkey Recep Tayyip Erdo an Training and Research Hospital, Mogadishu, Somalia; 3 Department of Emergency Medicine, Hatay Defne State Hospital, Hatay, Turkey

**Keywords:** empyema severity, biomarker, CALLY, SII, SIRI, PIV, HALP, empijem, težina bolesti, biomarkeri, CALLY, SII, SIRI, PIV, HALP

## Abstract

**Background:**

Empyema is an infectious disease characterised by the accumulation of pus in the pleural space, accompanied by inflammation. Patients often present with fever, chest pain, dyspnea, or cough, and delayed diagnosis or treatment is associated with a high mortality rate. Therefore, in this study, we aimed to investigate the clinical utility of the Systemic Inflammation Response Index (SIRI), Pan-Immune Value (PIV), C-Reactive Protein (CRP)-Albumin-Lymphocyte (CALLY) index, and the Systemic Immune-Inflammation Index (SII) to assess the severity of empyema and predict mortality risk.

**Methods:**

Seventy-three patients diagnosed with empyema in the thoracic surgery clinic between January 2022 and October 2024 were included in the study. Patients were categorised based on their need for intensive care. Demographic and laboratory data (white blood cell count, platelet count, albumin levels, pleural fluid culture results, and C-reactive protein levels) were recorded. Differences between empyema patient groups classified according to disease severity were analysed.

**Results:**

Among the 73 patients included in our study, four deaths were recorded. The CALLY index could be used for intensive care and ward triage (p= 0.039). Although the SII and SIRI indices were not suitable for triage purposes, the SIRI index was found to help predict mortality (p= 0.01).

**Conclusions:**

In empyema cases, the CALLY index can be used to determine the need for intensive care, whereas the SIRI can help predict mortality risk.

## Abstract (sr)

**Uvod:**

Empijem je infektivno oboljenje koje se karakteriše nakupljanjem gnoja u pleuralnom prostoru, praćeno izraženim inflamatornim odgovorom. Pacijenti se najčešće javljaju sa povišenom telesnom temperaturom, bolom u grudima, dispnejom ili kašljem, dok su odloženo postavljanje dijagnoze i započinjanje terapije povezani sa visokom stopom mortaliteta. Cilj ove studije bio je da se ispita klinička primenljivost indeksa sistemskog inflamatornog odgovora (SIRI), pan-imunog indeksa (PIV), indeksa C-reaktivni protein-albumin-limfociti (CALLY) i sistemskog imuno-inflamatornog indeksa (SII) u proceni težine empijema i predikciji rizika od smrtnog ishoda.

**Metode:**

U studiju je uključeno 73 pacijenata sa dijagnozom empijema, lečenih na Klinici za grudnu hirurgiju u periodu od januara 2022. do oktobra 2024. godine. Pacijenti su podeljeni u grupe na osnovu potrebe za lečenjem u jedinici intenzivne nege. Prikupljeni su demografski i laboratorijski podaci (broj leukocita, broj trombocita, nivo albumina, rezultati kulture pleuralnog eksudata i koncentracija C-reaktivnog proteina). Analizirane su razlike između grupa pacijenata klasifikovanih prema težini bolesti.

**Rezultati:**

Od ukupno 73 analizirana pacijenta, zabeležena su četiri smrtna ishoda. Utvrđeno je da se CALLY indeks može koristiti kao pomoćno sredstvo u trijaži pacijenata između odeljenja i jedinice intenzivne nege (p= 0,039). Iako indeksi SII i SIRI nisu pokazali značaj u trijaži, SIRI indeks je bio koristan u predviđanju mortaliteta (p= 0,01).

**Zaključak:**

Kod pacijenata sa empijemom, CALLY indeks može da posluži kao pokazatelj potrebe za intenzivnim lečenjem, dok SIRI indeks može da doprinese proceni rizika od smrtnog ishoda.

## Introduction

Empyema, characterised by the accumulation of pus in the pleural space, often causes parapneumonia. However, it can also arise from spinal infections, bronchogenic carcinoma, oesophageal rupture, bronchopleural fistula, trauma, or postsurgical complications [1][2]. Approximately 20% to 40% of hospitalised patients with pneumonia develop parapneumonic effusion, with 5% to 10% progressing to empyema, and approximately 15% of these patients succumb to the disease [2].

The differentiation between transudative and exudative pleural fluid was based on Light's criteria [3]. Empyema lasting less than four weeks is defined as acute empyema, whereas cases persisting longer are classified as chronic empyema [4]. The most common causative agents are gram-positive bacteria, particularly *Streptococcus* and *Staphylococcus*, while fungi are responsible for only 1-3% of cases [5].

Although empyema shares a clinical presentation with lung abscesses, it is associated with higher morbidity and mortality rates. The treatment approach differs owing to the early need for surgical intervention [6]. Contrast-enhanced thoracic CT remains the most reliable method for diagnosis and treatment planning [7].

Several indices have been used to define predictive values across various conditions. The Systemic Inflammation Response Index (SIRI) is determined by the neutrophil-to-lymphocyte and monocyte ratio (neu*mono/lym) and has predictive value for identifying metastatic cancers [8].

The Systemic Immune-Inflammation Index (SII) is calculated using the platelet-to-lymphocyte and neutrophil ratio (plt*neu/lym) and is used to predict mortality and prognosis in cancer patients [9].

The Pan-Immune Value (PIV) is determined by the ratio of neutrophils, platelets, and monocytes to lymphocytes (neupltmono/lym). It is used to assess the impact of the inflammatory response on prognosis in cancer patients [10].

The haemoglobin-albumin-lymphocyte-platelet (HALP) score is employed to monitor systemic effects and nutritional status in oncological patients, where inflammation plays a critical role in fundamental processes [11].

In this study, we aimed to investigate the utility of haematological and biochemical markers in predicting the need for intensive care and mortality in patients with empyema.

## Materials and methods

### Overview

This retrospective study was conducted at a tertiary education and research hospital after obtaining approval from the ethics committee. A total of 73 patients diagnosed with empyema between January 2022 and October 2024 in the thoracic surgery clinic were identified and included in the study through the hospital information management system.

Patients diagnosed with empyema under ICD-10 code *J86* in the hospital management system, as well as those diagnosed in consultation and discharge notes, were included. Patients aged <18 years, those with missing data, and those without a definitive empyema diagnosis were excluded.

### Variables

The variables in this study included haematological and biochemical markers (such as Systemic Inflammation Response Index (SIRI), Pan-Immune Value (PIV), C-Reactive Protein (CRP)-Albumin-Lymphocyte (CALLY) index, and the Systemic Immune-Inflammation Index (SII), and the haemoglobin-albumin-lymphocyte-platelet (HALP) scores as independent variables, ICU admission and mortality as dependent variables, and demographic and clinical factors (such as age, sex, and comorbidities) as control variables.

### Research design

This study employed a retrospective observational design and was conducted at a tertiary education and research hospital. This study aimed to investigate the clinical utility of haematological and biochemical markers in predicting the need for intensive care and mortality in patients diagnosed with empyema.

### Statistical analysis

The data obtained in this study were analysed using SPSS version 27. Graphs were created using GraphPad Prism, version 9. Descriptive statistics were obtained after the data processing. Categorical variables are expressed as percentages and frequencies, whereas numerical data were subjected to distribution analysis. Numerical data that followed a normal distribution were described as mean ± standard deviation, while those that did not follow a normal distribution were described as median and interquartile range. Chi-square tests were applied to categorical variables, and t-tests were used for numerical values that followed a normal distribution. Nonparametric tests were employed for numerical values that did not follow a normal distribution. To assess the effect of the independent variables, Receiver Operating Characteristic (ROC) analysis was performed, and sensitivity and specificity analyses were conducted based on the determined cut-off values. Statistical significance was set at p<0.05.

## Results

Among the 73 patients included in the study, mortality was not observed in 69 patients, while four patients died. No significant differences were found between groups in terms of sex, age, or comorbidities. A comparison of patient characteristics is presented in Table 1.

**Table 1:** Comparison of characteristic specification. (DM Diabetes mellitus (DM), hypertension (HT), chronic hepatitis B (HBV), chronic renal failure (CRF), congestive heart failure (CHF), intensive care unit (ICU), methicillin-sensitive Staphylococcus aureus (MSSA).

	Live (n = 69)	Ex (n=4)	p-value
Female (n, %)	48 (69.9)	4 (100)	0.094
Age (mean±SD)	32.07±17.02	41.5±28.86	0.142
Comorbidity (n)			
DM	6	1	0.915
HT	1	0	
Malignite	4	0	
HBV	3	0	
CRF	1	0	
CHF	3	0	
Tuberculosis (n, %)	17 (24.6)	2 (50)	0.276
Follow-up			
Service	51 (73.9)	0	0.007
ICU	18 (26.1)	4 (100)	
Culture			
Escherichia coli	9 (13.0)	1 (25)	0.226
Klebsiella	7 (10.1)	1 (25)	
Pseudomonas aeruginosa	2 (2.9)	1 (25)	
Enterococcus	1 (1.4)	0	
MSSA	1 (1.4)	0	

The mean platelet (PLT) level of the deceased patients was 147,750.00±45,573.20, while the mean PLT level of the surviving patients was 443,478.26±209,147.33. A significant decrease in PLT levels was observed in the deceased patients (p=0.040). A comparison of patients' biochemical values is presented in Table 2.

**Table 2:** Comparison of biochemical values. White Blood Cell (WBC), haemoglobin (HGB), neutrophil (NEU), leukocyte (LEU), monocyte (MONO), platelet (PLT), and C-Reactive Protein (CRP) levels

	Service	ICU	p-value	Live (n=69)	Ex (n=4)	p-value
WBC (mean±SD)	10.53±6.03	11.75±7.20	0.623	10.39±5.81	20.04±10.56	0.104
Hgb (mean±SD)	11.71±2.03	10.77±3.17	0.031	11.53±2.45	9.77±2.68	0.675
Neu (median (IQR))	6.27 (.45)	9.22 (9.17)	0.359	6.91 (8.59)	14.29 (16.23)	0.021
Leu (mean±SD)	1.39 (0.89)	1.53 (1.12)	0.972	1.83±1.81	1.42±1.06	0.929
Mono (mean±SD)	0.87±0.44	0.81±0.54	0.137	0.87±0.47	0.65±0.41	0.929
Plt (mean±SD)	439.19±196.28	404.13±249.64	0.028	443.47±209.14	147.75±45.57	0.040
Albumine (mean±SD)	3.18±0.59	2.8±0.57	0.658	3.08±0.61	2.85±0.50	0.231
CRP (mean±SD)	125.86±99.21	180.26±125.22	0.039	137.88±107.01	204.25±163.45	0.144

**Figure 1:**
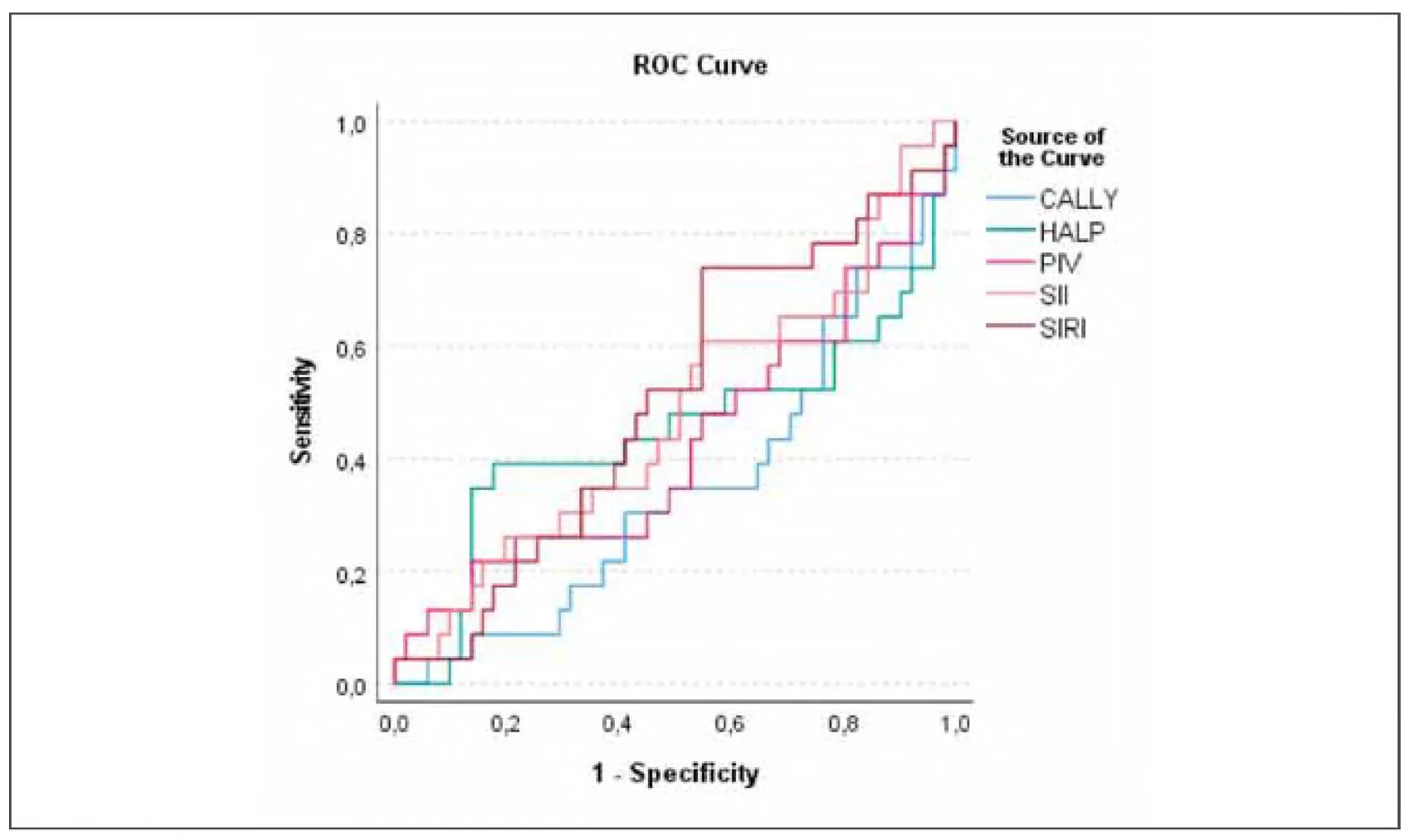
ROC analysis of follow-up.

Significant differences were observed in the performances of the different models in predicting mortality (Table 3). The SIRI model had the highest AUC value (0.703; p<0.05), demonstrating a better performance in predicting mortality than the other models. The HALP model also showed similarly strong performance (AUC=0.692; p<0.05). However, the AUC values of the SII, PIV, and CALLY models were close to 0.5, indicating their limited usefulness in predicting mortality. Table 4 and Figure 1 present the results of the ROC analysis.

**Table 3:** Comparison of haematological indices. Systemic Immune-Inflammatory Response Index (SIRI), Systemic Immune-Inflammation Index (SII), Pan-Immune Value (PIV), Haemoglobin-Albumin-Lymphocyte-Platelet Index (HALP), and C-Reactive Protein-Albumin-Lymphocyte Index (CALLY).

	Service	ICU	p-value	Live (n=69)	Ex (n=4)	p-value
SII (median (IQR))	2284.31<br>(2565.27)	1922.44<br>(3165.26)	0.784	1865,55<br>(2593,17)	2042.74<br>(4911.79)	1
SIRI (median (IQR))	4.28 (8.24)	4.335 (8.12)	0.930	4,07 (7.55)	7.31 (11.08)	0.186
PIV (median (IQR))	2147.25<br>(2445.66)	1124.78<br>(2870.25)	0.324	2130.62<br>(2685.53)	1069.33<br>(1106.44)	0.474
CALLY (median (IQR))	0.055 (0.09)	0.025 (0.06)	0.039	0,054 (0.07)	0.017 (0.30)	0,340
HALP (median (IQR))	0.145 (0.08)	0.101 (0.20)	0.444	0.11 (0.11)	0.28 (0.29)	0.212

**Table 4:** ROC analysis of mortality. Systemic Immune-Inflammatory Response Index( SIRI), Systemic Immune-Inflammation Index (SII), Pan-Immune Value (PIV), Haemoglobin-Albumin-Lymphocyte-Platelet Index (HALP), and C-Reactive Protein-Albumin-Lymphocyte Index (CALLY).

		AUC	p-value	Cut-off	Sensitivty	Specificity
SII	ICU	0.520	0.789	995.69	%30.4	%84.3
	Mortality	0.504	0.980	1894	%75	%50.7
SIRI	ICU	0.506	0.930	3.13	%73.9	%45.1
	Mortality	0.703	0.010	4.44	%100	%58
PIV	ICU	0.572	0.348	668.87	%39.1	%80.4
	Mortality	0.612	0.101	2113.04	%100	%50.3
CALLY	ICU	0.650	0.030	0.0454	%65.2	%64.7
	Mortality	0.649	0.415	0.019	%0	%98.6
HALP	ICU	0.556	0.500	0.0829	%47.8	%78.4
	Mortality	0.692	0280	0.246	%75	%84.1

**Figure 2:**
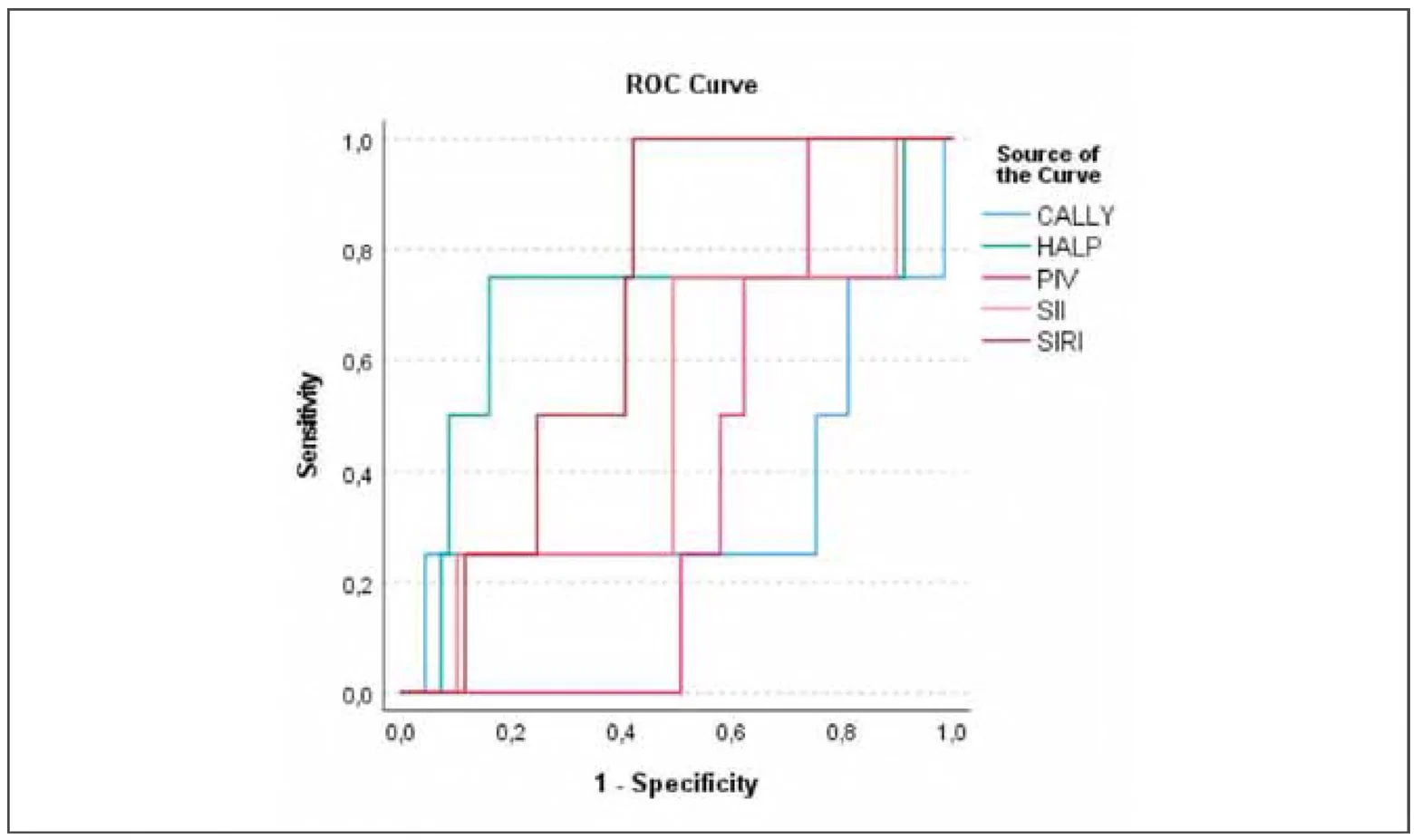
ROC analysis of mortality.

All deceased patients (100%) had gram-negative bacteria, while 90% of surviving patients showed gram-negative bacterial growth, with no significant difference between the groups (p=0.567). In the comparison of haematological indices based on Gram staining type, no significant differences were observed in the SII, SIRI, PIV, CALLY, and HALP scores. Table 5 presents a comparison of mortality and haematological indices based on Gram staining. Figure 2

**Table 5:** Comparison of mortality and haematological indices according to gram staining. (Systemic Immune-Inflammatory Response Index (SIRI), Systemic Immune-Inflammation Index (SII), Pan-Immune Value (PIV), Haemoglobin-Albumin-Lymphocyte-Platelet Index (HALP), and C-Reactive Protein-Albumin-Lymphocyte Index (CALLY).

	Gram		
	Positive (n=2)	Negative (n=21)	p-value
SII	3222.77±209.49	2832.09±2210.71	0.214
SIRI	4.49±2.84	6.68±5.62	0.228
PIV	3813.60±1566.84	2060.57±1587.35	0.905
CALLY	0,11 (-)	0.06 (0.50)	0.957
HALP	0.0059±0.004	0.166±0.154	0.140
Live	2 (%10)	18 (%90)	0.567
Ex	0	3 (%100)	

### Ethical approval

This study was conducted with the approval of the MSTH Ethics Committee, dated December 12, 2024, with approval number 20409. Data confidentiality was maintained throughout the study in accordance with the Helsinki Declaration criteria.

## Discussion

Empyema, which presents with mortal and morbid outcomes, has a low prevalence and requires early recognition and appropriate triage for timely and effective intervention based on its severity. In our study, indices derived from the initial results obtained during the first encounter with patients were used to ensure early diagnosis and intervention. The CALLY score, calculated using C-reactive Protein(CRP), albumin, and lymphocyte counts, was found to be significantly lower in intensive care unit patients, indicating its potential utility for triage purposes.

The CALLY score, an immunonutritional index derived from serum CRP albumin, and lymphocyte counts, has shown significant potential for determining disease severity in various cancers. In epithelial ovarian cancer (EOC), a CALLY index 3 is associated with overall survival (OS) and disease-free survival (DFS), highlighting its utility as a prognostic biomarker for postoperative outcomes [12]. Similarly, in nasopharyngeal carcinoma (NPC), integration of the CALLY score with Epstein-Barr virus (EBV) DNA levels enhances prognostic stratification, outperforming traditional TNM staging in predicting OS, distant metastasis-free survival (DMFS), and locoregional recurrence-free survival (LRRFS) [13]. In oral cavity squamous cell carcinoma (OSCC), a CALLY index below 0.65 was associated with increased pathological aggressiveness and reduced overall survival (OS) and disease-free survival (DFS), further supporting its role as an independent risk factor for poor prognosis [14]. Although studies on the CALLY score after percutaneous coronary intervention (PCI) in patients with coronary artery disease (CAD) are lacking, it has been proposed as a potential novel inflammatory biomarker influencing adverse outcomes [15]. In our study, the CALLY score provided valuable insight into disease severity by predicting the need for intensive care, with a sensitivity of 65.2% at a cut-off value of 0.045. The Systemic Immune-Inflammation Index (SII), which reflects the balance between inflammation and the immune response, is increasingly recognised as a significant predictor of mortality in various medical conditions. In patients with rheumatoid arthritis (RA), a higher SII is associated with a 1.48-fold increased risk of all-cause mortality and a 1.51-fold increased risk of cardiovascular mortality, highlighting its potential as a prognostic marker for these patients [16]. Similarly, in the context of ST-segment elevation myocardial infarction (STEMI), initial analyses suggested a relationship between high SII and mortality; however, after adjusting for confounding factors, such as creatinine and brain natriuretic peptide levels, these relationships were not significant [17]. In our study, no impact of the SII was observed on triage for intensive care or mortality.

The Systemic Inflammation Response Index (SIRI) has emerged as a significant predictor of mortality in various health conditions, including COVID-19, myocardial infarction, hyperuricemia, cancer, and chronic kidney disease. In COVID-19 patients, high SIRI levels are associated with increased disease severity and mortality, with a threshold value of 2.057, indicating a higher risk of mortality [18]. Similarly, in patients with ST-elevation myocardial infarction (STEMI), higher SIRI levels are associated with lower survival rates. However, these relationships are influenced by confounding factors such as creatinine and brain natriuretic peptide levels [17]. In individuals with hyperuricemia, the SIRI was independently associated with all-cause and cardiovascular mortality, indicating a significant increase in mortality risk [19]. Among cancer survivors, a high SIRI, especially when combined with sarcopenia, was associated with increased risks of all-cause, cancer-specific, and non-cancer-specific mortality [20]. In patients with chronic kidney disease, SIRI was an important independent risk factor for both cardiovascular and all-cause mortality, with a non-linear relationship observed, particularly in the early stages of the disease [21]. While no significant differences were found between groups in our comparison, an analysis using a threshold of 4.44 showed 100% sensitivity for mortality. As seen in the literature, the SIRI is an essential index for determining patient mortality.

### Limitations

Our study had several limitations. The foremost limitation was the retrospective nature of the study, which led to data loss. Conducting a prospective multicenter study would enhance the predictive power of the findings.

## Conclusion

In empyema cases, the CALLY index can be utilised to determine the need for intensive care, whereas the SIRI index may be helpful in assessing mortality risk. These indices provide valuable insights for clinical decision-making and patient management, offering potential tools for better prognosis and risk stratification in patients with empyema. Further research is needed to confirm their clinical applicability and to explore their roles in improving patient outcomes.

## Dodatak

### Funding

The study did not receive any funding.

### Conflict of interest statement

All the authors declare that they have no conflict of interest in this work.
